# Supplementary material for: Seminoma and Embryonal Carcinoma Footprints Identified by Analysis of Integrated Genome-Wide Epigenetic and Expression Profiles of Germ Cell Cancer Cell Lines
Source: PLoS One. 2014 Jun 2;9(6):e98330. doi: 10.1371/journal.pone.0098330 (PMC4041891; doi:10.1371/journal.pone.0098330)
Supplement: File S1 — ZIP file containing DMRforPairs output for significant regions. Please start from the html files. (ZIP) [file pone.0098330.s008.zip › figures/27998.pdf]

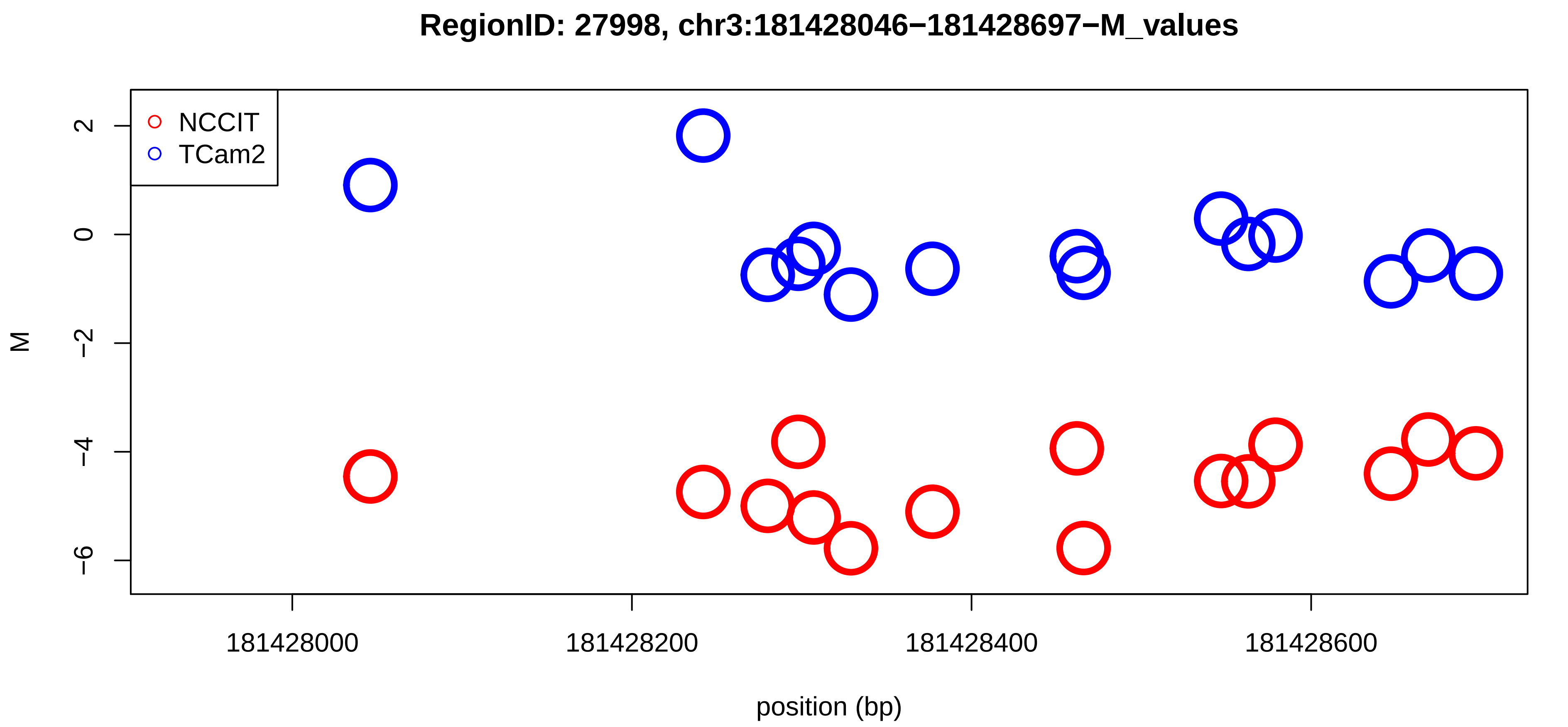
RegionID: 27998, chr3:181428046−181428697−M_values
NCCIT
TCam2
M
2
0
−2
−4
−6
181428000
181428200
181428400
181428600
position (bp)

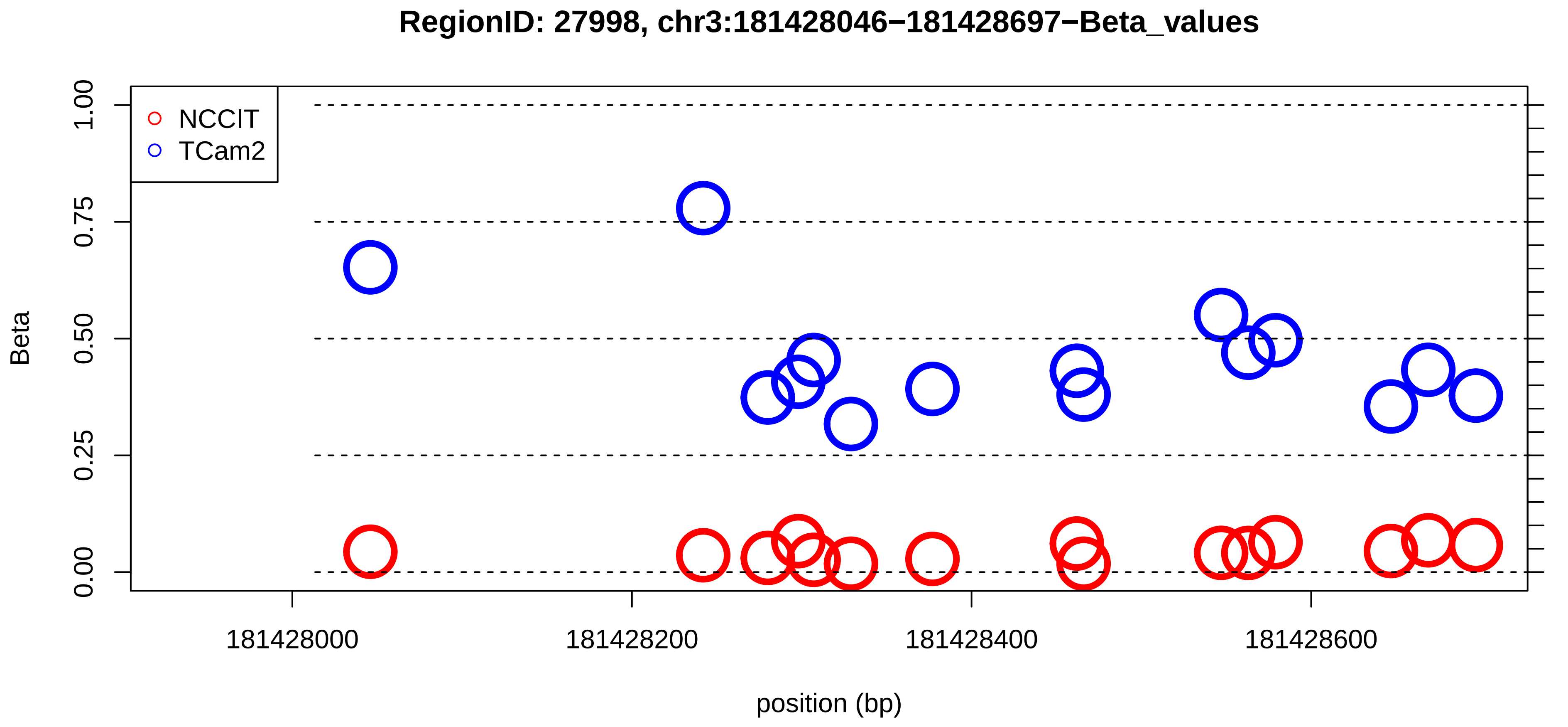
RegionID: 27998, chr3:181428046−181428697−Beta_values
NCCIT
TCam2
Beta
1.00
0.75
0.50
0.25
0.00
181428000
181428200
181428400
181428600
position (bp)

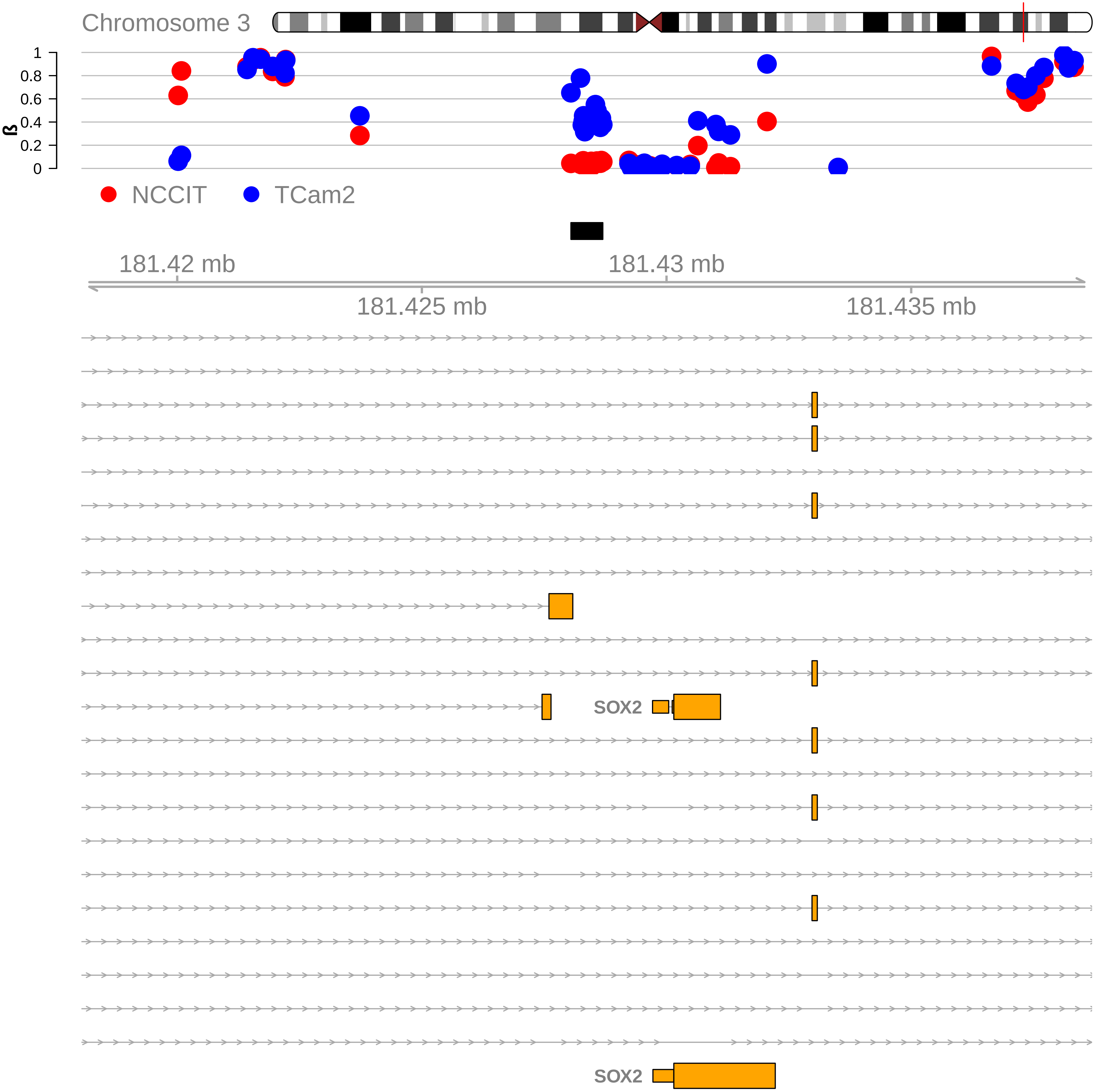

Chromosome 3
ß
1
0.8
0.6
0.4
0.2
0
NCCIT
TCam2
181.42 mb
181.425 mb
181.43 mb
181.435 mb
SOX2
SOX2
